# Removal of Non-Steroidal Anti-Inflammatory Drugs from Drinking Water Sources by GO-SWCNT Buckypapers

**DOI:** 10.3390/molecules27227674

**Published:** 2022-11-08

**Authors:** Mariafrancesca Baratta, Antonio Tursi, Manuela Curcio, Giuseppe Cirillo, Aleksey Vladimirovich Nezhdanov, Alexandr Ivanovic Mashin, Fiore Pasquale Nicoletta, Giovanni De Filpo

**Affiliations:** 1 Department of Chemistry and Chemical Technologies, University of Calabria, 87036 Rende, Italy; 2 Department of Pharmacy, Health and Nutritional Sciences, University of Calabria, 87036 Rende, Italy; 3 Applied Physics & Microelectronics, Lobachevsky State University of Nizhni Novgorod, Nizhni Novgorod 603105, Russia

**Keywords:** single-walled carbon nanotubes, graphene oxide, buckypaper, non-steroidal anti-inflammatory drugs, water sources, adsorption

## Abstract

Pharmaceutical products such as antibiotics, analgesics, steroids, and non-steroidal anti-inflammatory drugs (NSAIDs) are new emerging pollutants, often present in wastewater, potentially able to contaminate drinking water resources. Adsorption is considered the cheapest and most effective technique for the removal of pollutants from water, and, recently, membranes obtained by wet filtration method of SWCNT aqueous solutions (SWCNT buckypapers, SWCNT BPs) have been proposed as self-standing porous adsorbents. In this paper, the ability of graphene oxide/single-walled carbon nanotube composite membranes (GO-SWCNT BPs) to remove some important NSAIDs, namely Diclofenac, Ketoprofen, and Naproxen, was investigated at different pH conditions (pH 4, 6, and 8), graphene oxide amount (0, 20, 40, 60, and 75 wt.%), and initial NSAIDs concentration (1, 10, and 50 ppm). For the same experimental conditions, the adsorption capacities were found to strongly depend on the graphene oxide content. The best results were obtained for 75 wt.% graphene oxide with an adsorption capacity of 118 ± 2 mg g^−1^ for Diclofenac, 116 ± 2 mg g^−1^ for Ketoprofen, and 126 ± 3 mg g^−1^ for Naproxen at pH 4. Overall, the reported data suggest that GO-SWCNT BPs can represent a promising tool for a cheap and fast removal of NSAIDs from drinking water resources, with easy recovery and reusability features.

## 1. Introduction

Emerging pollutant is a general term labeling compounds recently resulting as dangerous for both environment and human health, with an enhanced threat to humans being represented by their increased concentration in water sources and the lack of legal limits at national or international levels.

Nowadays, due to their widespread use to prevent and treat human diseases, pharmaceutical compounds (PCs) represent a new class of emerging pollutants [1]. Their improper disposal and accidental contaminations [2], as well as their excretion and dispersion in wastewater after their assumption, could carry out to their accumulation in fresh water resources. Due to their low molecular weight, polar nature, and hydrophilicity, PCs are not separated by conventional wastewater treatment plants, and thus their persistence and accumulation can cause unforeseen effects on the environment [3,4,5,6,7]. PCs can be detected in aquatic environments around the world [8,9] with concentrations up to several mg L^−1^ as a consequence of their long-term stability [10,11,12]. In addition, national drinking-water directives generally do not define limits to the PC presence, in spite of any precautionary principle claiming for a complete removal of such pollutants since they can cause acute toxicity, including genotoxicity, endocrine disruption, development of pathogen resistance, and other often unknown effects on public health and environment [13,14].

Non-steroidal anti-inflammatory drugs (NSAIDs) represent the most important class of the new emerging pollutants due to their wide use as analgesic, anti-inflammatory, antipyretic, and pain-relief drugs [15]. Diclofenac (DIC), Ketoprofen (KET), and Naproxen (NAP) are some of the main worldwide used NSAIDs, with a production of many hundreds of tons per year, which can be found at huge concentrations in wastewater, surface water, groundwater, and drinking-water [16].

In the last years, researchers have proposed several technologies for an effective PC removal, including bio-treatment [17], advanced oxidation [18,19], membrane filtration [20,21], and ozonation [22]. Nevertheless, these processes are affected by some drawbacks, such as high costs, toxic by-products, slow and poor removal efficiencies.

Among others, adsorption is considered the cheapest, easiest, and most effective technique for pollutant removal from water with no formation of toxic by-products [23,24]. Several porous materials have been proposed as efficient adsorbents for pharmaceutical compounds thanks to their stable chemical structure, high porosity, easy functionalization, and large adsorption capacities [25,26]. In particular, activated carbons [27,28], waste materials [29,30], metal-organic frameworks (MOFs) [31], magnetic nanoparticles [32,33], single- and multi-walled carbon nanotubes (SWCNTs and MWCNTs) [34,35], and graphene oxide sheets (GO) have been tested for the removal of NSAIDs over the last years [36]. 

In particular, carbon-based materials were widely explored as promising adsorbents due to their superior surface properties, although they suffer from difficult recovery and reuse as a consequence of their powder consistency. More recently, several research groups have assembled powder adsorbents in porous membranes by either using polymer additives or exploiting the self-assembling properties of some materials [37,38,39]. Buckypapers (BPs) are self-standing porous membranes obtained by self-assembling SWCNT and/or MWCNT bundles mainly via π-π and van der Waals interactions during the wet filtration of carbon nanotube solutions [40,41]. BPs are characterized by interesting rheological, thermal, and electrical properties [42] and are successfully applied in filtration and adsorption processes. In fact, they show porous structure, low density, easy functionalization, as well as the possibility to host other adsorbents, such as natural polymers [43,44], MOF [45,46], and GO [47,48], to increase the adsorption efficiencies [46,49].

In our previous work, we proved that the partial substitution of SWCNTs with GO in BPs structure allowed the obtainment of self-standing hybrid buckypapers (GO-SWCNT BPs) useful for heavy metal removal with good efficiency [47]. With the present investigation, we aim to test the versatility of these GO-SWCNT BPs, evaluating the possibility of using them as a tool for the removal of NSAIDs (namely DIC, KET, and NAP) from wastewater. In detail, we extensively investigated the effect of key parameters (e.g., different GO wt. contents, pH values, and initial NSAID concentration) to determine the optimal adsorption conditions. Overall, we found that the adsorption capacity towards all NSAIDs increased with the graphene oxide percentage present in the membrane composition, proving the importance of the GO in the membrane composition.

## 2. Results

**Figure 1:**
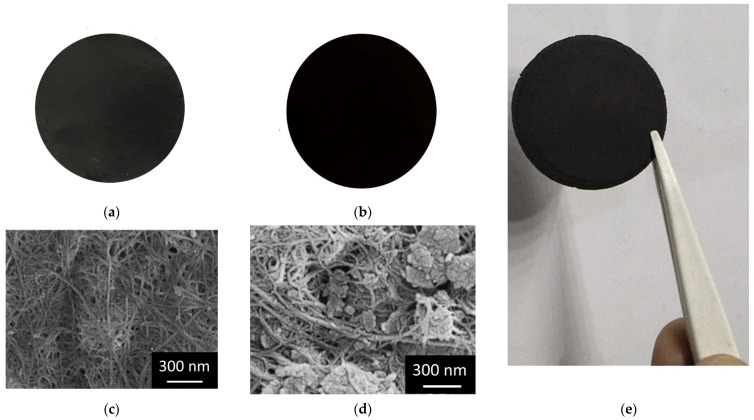
Pictures of (**a**) SWCNT BP and (**b**) 75% GO-SWCNT BP. SEM images of (**c**) SWCNT BP and (**d**) 75% GO-SWCNT BP. The presence of GO flakes embedded in the SWCNT BP network is evident in the latter image. Samples up to 75 wt.% GO were self-sustainable and flexible disks (**e**).

The wet method is probably the easiest procedure to obtain carbon nanotube buckypapers with homogeneous morphology [41]. Thus, SWCNT BPs were prepared according to this method by dispersing the SWCNT mixture (SWCNTs and carboxylic acid functionalized SWCNTs in the wt. ratio 2:1) in a TRITON-X100 water solution followed by vacuum filtration through a PTFE 5 μm porous filter. BP membranes were then washed with methanol several times, dried, and, finally, peeled off from the PTFE filter. GO-SWCNT BPs were prepared similarly to the procedure outlined for pure SWCNT BPs, with the substitution of some percentage (20, 40, 60, and 75 wt.%) of SWCNT mixture with GO (see Section 3 for more details). For all tested wt. ratios, self-standing and flexible BP disks were obtained for a GO loading up to 75 wt.% (Figure 1e), while a further increase in GO amount gave cracked films due to insufficient π-π and van der Waals interaction forces between SWCNT and GO. Such results agree with the decrease in the rheological performance (reduced values of tensile strength and fracture strain) reported in the literature when large GO amounts were used in GO-SWCNT BPs preparation [47].

As reported in Figure 1a,b, BPs looked very similar to the eye independently from the amount of GO present in the BP composition, being black and stable membranes with an average thickness of around 100 ± 2 µm and an average diameter of 38 ± 1 mm. The SEM investigations showed that the SWCNT BP morphology was characterized by the presence of the typical SWCNT clusters and bundles arising from van der Waals and π-π interactions, Figure 1c. It is also evident that the typical large porosity confers high permeability and large contact surface area to BPs, thus allowing for an effective adsorption process. At a microscopic level, the substitution of GO in GO-SWCNT BPs resulted in the presence of GO sheets homogenously hosted inside the SWCNT BP membranes, Figure 1d.

The NSAID removal properties by SWCNT and GO-SWCNT membranes were evaluated through adsorption experiments performed on water solutions of the three most used active compounds (Diclofenac, Ketoprofen, and Naproxen) at three different pH values (4, 6, and 8) and initial concentrations (1, 10, and 50 ppm). 

**Figure 2:**
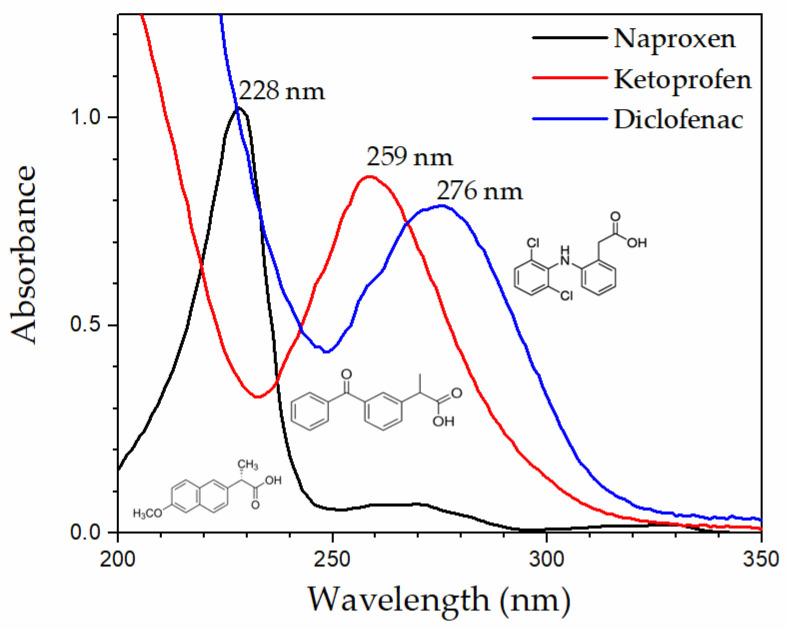
UV-Vis adsorption spectra and chemical structure of NAP, KET, and DIC.

In more detail, membrane disks were immersed in 200 mL beakers with a given NSAID water solution, and the kinetic profiles of drug recovery in the 0–72 h interval were recorded. The recovery percentage was spectro-photochemically obtained by measuring the absorbance of the three NSAIDs at their maximum absorption wavelength (276, 259, and 228 nm for DIC, KET, and NAP, respectively), with Figure 2 showing the UV-Vis adsorption spectra and chemical structure of the three drugs.

The recovery percentage, *Re*(%), was calculated according to the following Equation (1):

(1) $$Re\left(\%\right)=\frac{Abs_{0}-Abs_{t}}{Abs_{0}}\times 100$$

where *Abs*_0_ and *Abs_t_* were the drug absorbance at time 0 and *t*, respectively. 

**Figure 3:**
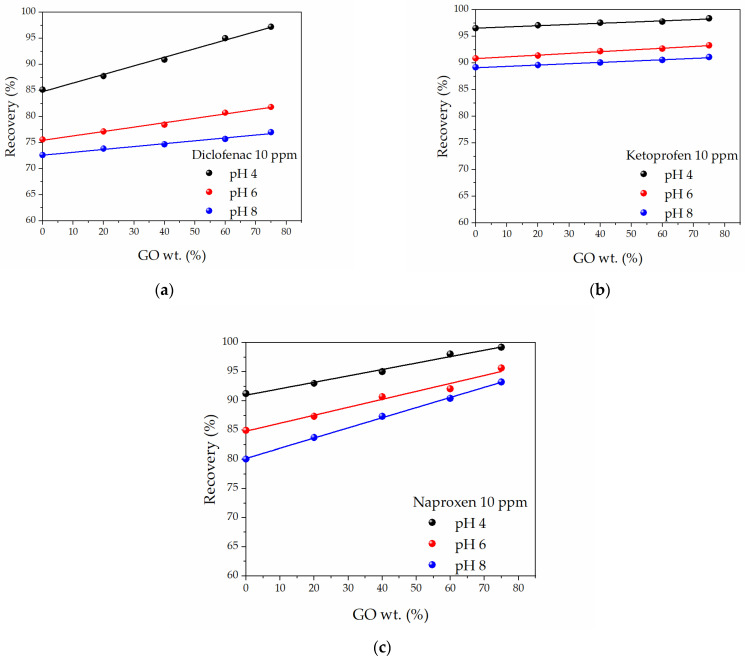
The recovery percentage, *Re*(%), of: (**a**) Diclofenac, (**b**) Ketoprofen, and (**c**) Naproxen water solutions at different pH values and 10 ppm initial concentration as a function of GO content in GO-SWCNT BPs.

Figure 3 shows the *Re*(%) behavior for the three NSAIDs in water solutions at different pH values and a 10 ppm initial concentration when GO-SWCNT BPs with increasing GO content were used as adsorbents.

The recovery of all NSAIDs as a function of pH after 72 h showed a similar trend with increasing adsorptions found when GO-SWCNT BPs with larger amounts of GO were used. pH 4.0 was the pH value ensuring the largest adsorption for all NSAIDs and GO-SWCNT BPs with the largest GO substitution (75 wt.%) granted the highest recovery values. 

In detail, neat SWCNT BPs showed the lowest recovery values (85.16, 96.53, and 91.23% @ pH 4 for DIC, KET, and NAP, respectively), which increased more or less linearly (*R*^2^ values always larger than 0.978) up to 97.2, 98.36, and 99.18% @ pH 4 for 75% GO-SWCNT BPs. Consequently, pH 4 was chosen as the pH value for carrying out the subsequent adsorption experiments. It is worth noting that the recovery could be increased up to the desired value (in accordance with the precautionary principles) also by successive adsorption steps with new or regenerated BPs (see infra).

**Figure 4:**
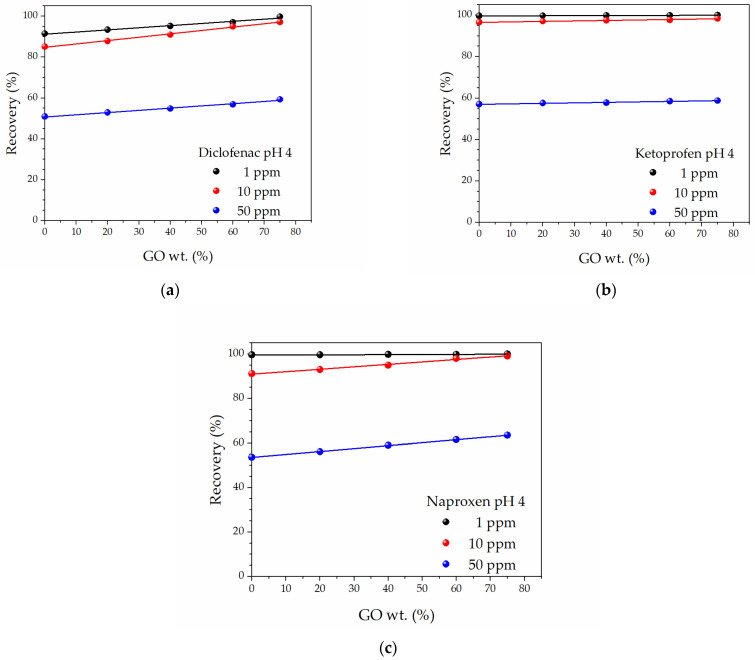
Recovery percentages, *Re*(%), at pH 4 as a function of GO content in GO-SWCNT BPs for: (**a**) Diclofenac, (**b**) Ketoprofen, and (**c**) Naproxen water solutions at different initial concentrations.

At pH 4, all membranes were able to recover the major amount of the NSAIDs present in the 1 and 10 ppm solutions (*Re*% larger than 85% in all cases), and the recovery was further increased when membranes with larger GO amounts were used (Figure 4). In these conditions, values larger than 97.2% were obtained when 75% GO-SWCNT BPs were used, confirming the positive effect of GO substitution. At a larger initial concentration (50 ppm), all *Re*(%) were lowered to values ranging from 50 to 64%, with the lowest adsorption value obtained from neat SWCNT BPs (50.91, 57.02, and 53.67% for DIC, KET, and NAP, respectively) and the highest from 75% GO-SWCNT BPs (59.28, 58.75, and 63.54% for DIC, KET, and NAP, respectively).

Such results gave an NSAID experimental SWCNT BPs adsorption capacity per unit of adsorbent mass, *q_exp_* (mg g^−1^), of 102 ± 2, 114 ± 2, and 108 ± 2 mg g^−1^, in the case of DIC, KET, and NAP 50 ppm aqueous solutions. *q_exp_* was defined as follows (Equation (2)):

(2) $$q_{exp}=\frac{m\left(NSAID\right)}{m\left(membrane\right)}$$

where *m*(*NSAID*) was the adsorbed NSAID mass in milligrams, and *m*(*membrane*) was the SWCNT BP or GO-SWCNT BP mass in grams.

The *q_exp_* values increased up to 118 ± 2 (+18%), 116 ± 2 (+2%), and 126 ± 3 mg g^−1^ (+16%) for the adsorption of DIC, KET, and NAP 50 ppm aqueous solutions by 75% GO-SWCNT BPs, confirming once again the best adsorption capacity of 75% GO-SWCNT BPs. The obtained *q_exp_* values were higher than those obtained for the adsorption of DIC by rGO flakes (34.1 mg g^−1^) [50], for DIC and NAP removal by some -COOH multi-walled carbon nanotubes (*q_exp_* around 40 mg g^−1^) [51], or for NAP removal by MOF powders (66.1 mg g^−1^) (MIL-101-NO_2_) [52]. Even if particular GO and MOF samples with comparable NSAID recovery values can be found in the literature [52,53,54,55,56], it is worth noting that GO-SWCNT BPs are self-standing films, which can be more easily used, recovered, and regenerated than any powder-like adsorbent (see infra).

Adsorption, including both chemisorption (involving ionic or covalent bonds) and physisorption (van der Waals forces), is a surface phenomenon where drug molecules accumulate at the interface between an adsorbent and a fluid solution of adsorbate [57]. Generally, the adsorption mechanism is dependent on the chemical nature of active compounds, the adsorbent properties (including chemical nature, number of active sites present, water wettability, and porosity), and the experimental conditions (e.g., initial concentration, ionic strength, pH, temperature, and contact time). Since the same experimental conditions were used for all BPs, the differences in adsorption can arise from their different physico-chemical characteristics. From previous studies [47], we know that the GO substitution in SWCNT BPs causes both a decrease in membrane porosity from around 74 ± 5% (neat SWCNT BP) to around 41 ± 5% (75% GO-SWCNT BP) and a decrease in the water contact angle from 71.3 ± 0.5° (neat SWCNT BP) to 41.2 ± 0.5° (75% GO-SWCNT BP). In addition, we found that the specific surface area changed from a value of 185 ± 20 m^2^ g^−1^ (neat SWCNT BP) to 126 ± 15 m^2^ g^−1^ (75% GO-SWCNT BP).

On the basis of the available datasheets of SWCNTs, COOH-SWCNTs, and GO, indicating a -COOH group percentage in COOH-SWCNTs of around 1%, and a 4.8% oxidation at the GO edge, it is possible to speculate that the NSAID adsorption increase with GO amount could be attributed to the presence of a larger number of oxygen groups onto the BP surfaces, as it is known that they favor the adsorption of NSAID molecules by the formation of H-bonding [51,58]. Such chemical functionalities act as active sites for the NSAID capture and are able, at the same time, to improve surface wettability, thus compensating for the detrimental porosity reduction in GO-SWCNT BPs due to the better packaging of GO sheets.

**Figure 5:**
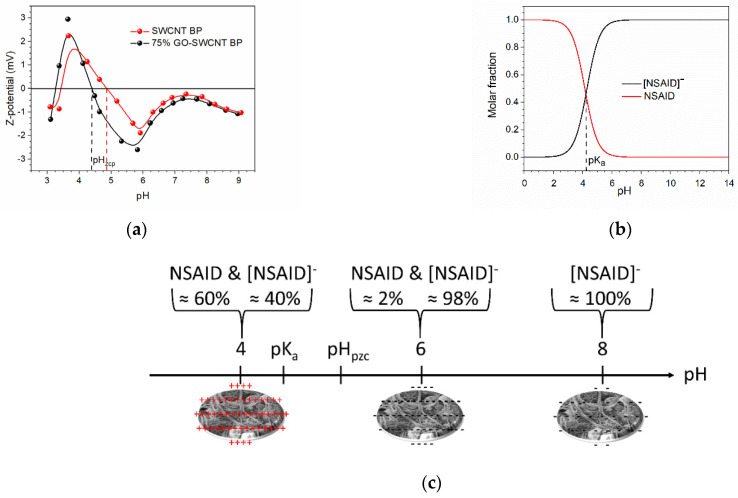
(**a**) Z-potential curves of an SWCNT BP (pH_zcp_ ≈ 4.86) and 75% GO-SWCNT BP (pH_zcp_ ≈ 4.41). The lines are just a guide for the eye. (**b**) Typical speciation diagram determined by the mass law equation for an NSAID. DIC, KET, and NAP are characterized by a pK_a_ value of 4.15, 4.30, and 4.15, respectively, as reported in [60]. (**c**) Naïve representation of NSAID and NSAID conjugates base ([*NSAID*]^−^) molar fractions and charge present on BPs at the investigated pH values (4, 6, and 8).

Z-potential measurements, Figure 5a, showed that the pH_zcp_ (the pH value at which the net charge onto the membrane surface is zero) is ≈4.86 and ≈4.41 for SWCNT BPs and 75% GO-SWCNT BPs, respectively. Consequently, BPs possess a net positive surface charge at pH 4, becoming negative at pH 6 and 8 (Z-potential @ pH 8 < Z-potential @ pH 6). 

The Z-potential of both membranes becomes more negative at pH 6 due to the ionization of the -COOH groups present on the SWNTs, most probably increase further as a result of the conformational changes of the nanodomains with pH [59]. In addition, when the pH of the solution exceeds the pK_a_ (4.15, 4.30, and 4.15 for DIC, KET, and NAP, respectively [60]), the active compounds dissociate more easily into their anionic forms. From the speciation diagram determined by the mass law equation (Figure 5b), the investigated NSAID molecules resulted in being in part negatively charged (molar fraction ≈ 40%) at pH 4, and almost all negatively charged at pH 6 and 8 (molar fraction ≈ 98 and ≈100%, respectively). These conditions made the NSAID adsorption onto BPs more favorable at pH 4 because of the presence of attractive electrostatic interactions between the anionic form of NSAID and the positively charged adsorbents and less favorable at pH 6 and 8, where electrostatic repulsions are expected to occur between negatively charged adsorbate and adsorbent, Figure 5c. Even if the BP surface charge at pH 8 is less negative than that at pH 6, the adsorption at the higher pH value could be hindered by the competition among a larger number of negatively charged adsorbent molecules. Therefore, the significant role of electrostatic interactions in the NSAID adsorption process by BPs is evident. The observed decrease of adsorption as a function of pH value agrees with the results previously reported in the literature [61,62]. Nevertheless, other adsorption processes, such as π-π interaction/stacking between SWCNT, GO and NSAID aromatic rings, as well as H-bonds, dispersive forces, and pore filling are expected to play a significant role in the adsorption of active compounds over BPs, accounting for the important recovery amount found at the different pH values [63].

**Figure 6:**
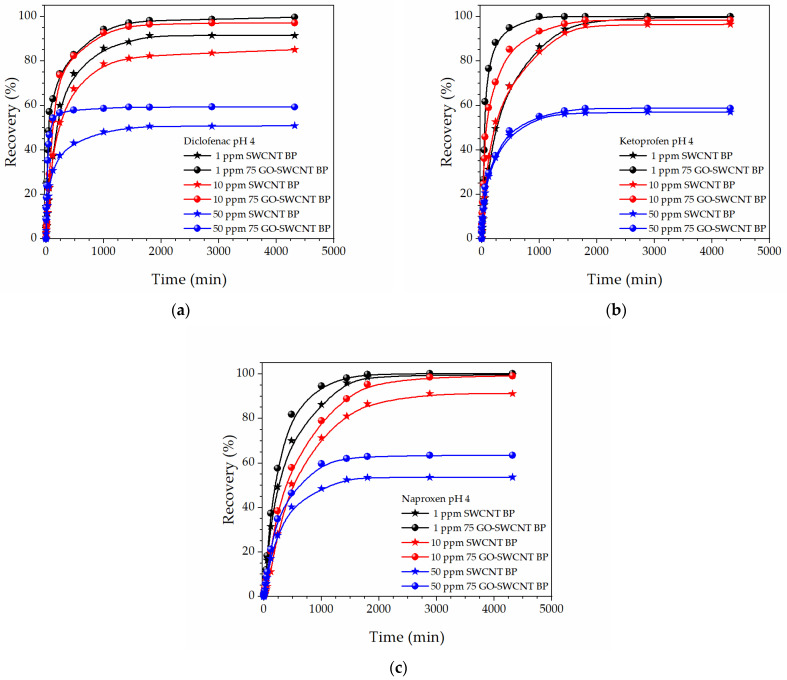
The time-dependent recovery percentage, *Re*(%), of: (**a**) Diclofenac, (**b**) Ketoprofen, and (**c**) Naproxen water solutions at pH 4 and different initial concentrations (1, 10, 50 ppm) for neat SWCNT and 75% GO-SWCNT BPs.

In order to follow the adsorption of NSAIDs as a function of time at different concentrations (1, 10, and 50 ppm) by neat SWCNT and 75% GO-SWCNT BPs, kinetic experiments were also performed (Figure 6). Experimental data were fitted by a non-linear optimization method [64] with a pseudo-first-order or a pseudo-second-order equation.

As per results reported in Table 1, the modeling indicated that the adsorption experimental data at 50 ppm were well fitted by the pseudo-second-order model compared to the pseudo-first-order as per their larger *R*^2^ (greater than 0.993), in agreement with the fittings obtained for NSAIDs adsorption by other adsorbents [56]. 

For all investigated NSAIDs, the pseudo-second-order adsorption rate constant, *k*_2_, increases when SWCNT BPs (0.26 ± 0.04, 1.4 ± 0.3, and 5.3 ± 1.1 × 10^−3^ g mg^−1^ min^−1^, for DIC, KET, and NAP, respectively) are replaced by GO-SWCNT BPs (0.84 ± 0.18, 1.5 ± 0.3, and 5.7 ± 1.4 × 10^−3^ g mg^−1^ min^−1^, for DIC, KET, and NAP, respectively). The found adsorption rate constants are of the same order and magnitude as those recently obtained for the NSAID adsorption from water by an aluminum-based metal-organic framework, MIL-53, (3.7 and 5.6 × 10^−3^ g mg^−1^ min^−1^ for DIC and NAP, respectively) [61] and by biochar samples [63], but lower than the adsorption rate constant for DIC adsorption by GO nanosheets (≈10 × 10^−3^ g mg^−1^ min^−1^) [50,52].

Regeneration and performance stability are important items for any adsorbent in view of potential industrial applications. The adsorption performance of 75% GO-SWCNT BP was measured at pH 4 in a 10 ppm DIC solution after four regeneration cycles, performed by soaking the membranes in ethanol or 24 h in order to favor the release of the adsorbed NSAID. The initial DIC recovery after the first immersion of a 75% GO-SWCNT BP was 97.2%, decreasing to 93.5% after the fourth regeneration cycle, confirming the reusability and stability of GO-SWCNT BP in industrial wastewater applications. In addition, GO-SWCNT BP membranes offer the opportunity for easy scalability as filtration units both in parallel for a large-scale treatment and in series for an increase in the adsorption efficiency.

## 3. Materials and Methods

### 3.1. Buckypaper Preparation and Characterization

SWCNT BPs were prepared according to the wet method procedure reported in the literature [47] by using mixtures of commercially available single-walled carbon nanotubes, SWCNTs, (length > 5 µm, and average diameter of 1.4 ± 0.1 nm) and carboxylic acid functionalized SWCNTs (COOH-SWCNTs, 1.00% carboxylic acid, 0.5 < average bundle length < 1.5 µm, and 4 < diameter < 5 nm, as reported in their datasheets from Merck/Sigma Aldrich, Darmstadt, Germany). Briefly, after the dispersion of 50 mg SWCNT and COOH-SWCNT mixtures (wt. ratio 2:1) in a surfactant water solution (250 mL 0.4% TRITON X100) by an ultrasonic bath (M1800H-E, Bransonic, Danbury, CT, USA) for 30 min, the solutions were filtered through poly(tetrafluoroethylene disks (PTFE, diameter = 47 mm, average pore size = 5 µm, Durapore©, Merck, Darmstadt, Germany) by a vacuum pump (pressure = −0.04 bar). Then, BPs were washed several times with methanol, dried at room temperature, and, lastly, peeled off from the PTFE filter.

Single-walled carbon nanotube/graphene oxide buckypapers, GO-SWCNT BPs, were similarly prepared after the substitution of a given weight amount (20, 40, 60, and 75 wt.%) of the SWCNT mixture with an identical quantity of graphene oxide (GO, 15–20 sheets, 4.8% edge-oxidized, Merck/Sigma Aldrich, Darmstadt, Germany). As shown in Figure 1, the wet method gave BPs the aspect of flexible self-standing membranes (average thickness 100 ± 2 µm and average diameter 38 ± 1 mm). The maximum GO loading in GO-SWCNT BPs was limited at 75 wt.% as cracked films were obtained for larger GO amounts. All chemicals were reagent grade and purchased from Merck/Sigma Aldrich, Darmstadt, Germany.

The morphology of buckypapers was investigated by scanning electron microscopy (LEO 420, Leica Microsystems, Cambridge, UK, accelerating voltage of 10 kV) after their sputtering with an ultrathin gold layer.

The surface area of buckypapers was obtained from the N_2_ adsorption isotherms at 77 K by a surface area analyzer (Belsorp Mini X, MicrotracBEL, Osaka, Japan) via the Brunauer-Emmett-Teller method. The surface charge of buckypapers was measured by a zeta potential analyzer (SurPASS TM 3, Anton Paar Italia S.R.L., Turin, Italy, equipped with an adjustable gap cell) as a function of pH value (from about pH 9 to pH 3 @ T = 25 °C). A pair of each membrane with a cross-section of 2 × 1 cm^2^ was mounted on the sample holders. The changes in pH were achieved by the addition of 0.05 M HCl, and the zeta potential was calculated from streaming potential measurements using the equation by Helmholtz and Smoluchowski [65].

### 3.2. NSAID Adsorption by Buckypapers

The conditions used for the NSAID adsorption by SWCNT and GO-SWCNT buckypapers were as follows: Buckypaper mass 50 mg, NSAID solution volume 200 mL, NSAID concentration 1, 10, and 50 ppm, contact time 0–4320 min, temperature 25 °C, pH 4, 6, and 8. NSAID concentration was determined by UV-Vis analyses on an Evolution 201 spectrophotometer (ThermoFisher Scientific, Hillsboro, OR, USA) operating with 1.0 cm quartz cells by using the calibration curves of DIC, KET, and NAP. Ultrapure deionized water (18.3 MΩ cm, Arioso, Human Corporation, Korea) was used for the preparation of the aqueous solution after filtration by a 0.45 μm filter (Millex Syringe Filter, Merck, Darmstadt, Germany). 

The concentrations of undissociated NSAIDs and their conjugated base, [*NSAID*]^−^, as a function of pH were calculated with the following Henderson-Hasselbalch Equation (3):

(3) $$pH=pK_{a}+log\frac{{\left[NSAID\right]}^{-}}{NSAID}$$

The speciation diagram clearly indicates that around 60% of all NSAID molecules in the aqueous solution were in a neutral state at pH < 4, Figure 5b.

Kinetic experiments were performed in order to follow the adsorption of NSAIDs as a function of time at different concentrations (1, 10, and 50 ppm) by neat SWCNT and 75% GO-SWCNT BPs. 

It is known from the literature [53] that the rate equation for NSAIDS adsorption capacity per unit of adsorbent mass (mg g^−1^), *q_t_*, by carbonaceous adsorbents can follow either the Lagergren first-order Equation (4) or a pseudo-second-order Equation (5):

(4) $$\frac{dq_{t}}{dt}=k_{1}\left(q_{e}-q_{t}\right)$$

(5) $$\frac{dq_{t}}{dt}=k_{2}{\left(q_{e}-q_{t}\right)}^{2}$$

where *k*_1_ (min^−1^) and *k*_2_ (g mg^−1^ min^−1^) are the pseudo-first and pseudo-second order adsorption rate constants, *q_e_* is the NSAID adsorption capacity per unit of adsorbent mass (mg g^−1^) at equilibrium, respectively. The experimental concentrations measured as a function of time, *C*(*t*), were used to determine the experimental amount, *q_exp_*(*t*), (mg g^−1^) of NSAID adsorbed by BPs, according to the following Equation (6):

(6) $$q_{exp}\left(t\right)=\frac{C_{0}-C\left(t\right)}{m}\times V$$

where *C*_0_ and *C*(*t*) are the NSAID concentration in the solution at time zero and *t*, respectively. *V* is the volume of NSAID solution, and *m* is the mass of BPs.

Experimental data were fitted by a non-linear optimization method [64] with the following Equations (7) and (8), respectively:

(7) $$q_{t}=q_{e}\left(1-e^{-k_{1}t}\right)$$

and

(8) $$q_{t}=\frac{k_{2}q_{e}^{2}t}{1+k_{2}q_{e}t}$$

which can be easily obtained from the integration of Equations (4) and (5).

All measurements were done in triplicate, and data were expressed as means ± SD. The kinetics parameters were calculated by OriginPro 2019 Software (OriginLab Corporation, Northampton, MA, USA).

## 4. Conclusions

In this work, GO-SWCNT BPs were tested as adsorbent membranes of non-steroidal anti-inflammatory drugs, such as Diclofenac, Ketoprofen, and Naproxen. BPs were prepared with increasing GO wt. contents and their adsorption capacity was measured at different pH values and drug initial concentrations. The maximum NSAID removal was obtained at pH 4 and increased with a larger GO amount. For solutions with a drug concentration of 1 and 10 ppm, 75% GO-SWCNT BPs were able to remarkably reduce the NSAID amount, with a recovery always higher than 97%, while in the case of 50 ppm solutions, the adsorption capacity increased up to 118, 116, and 126 mg g^−1^ for DIC, KET, and NAP, respectively. The adsorption efficiency was maintained over five adsorption/four regeneration cycles without any important reduction (less than 4%). 

This paper confirms that the partial substitution of SWCNTs with GO can increase the adsorption properties of SWCNT BPs towards NSAIDs without affecting their mechanical properties. As a consequence, they can still be used as flexible and self-standing membranes for a cheap and fast removal of NSAIDs from drinking water resources and allow an easier recovery and reuse than powder-like adsorbents can. Finally, it should be emphasized the importance of the easy scalability of the proposed adsorption membranes both in series for an efficiency increase and in parallel for applications in large-scale wastewater treatment plants. Overall, the experimental evidence here reported well addressed the precautionary principles towards the environment and human health, suggesting the adoption of adequate wastewater treatment techniques able to ensure the maximal removal of emerging pollutants, including non-steroidal anti-inflammatory drugs.

## Figures and Tables

**Table 1:** Pseudo-first and pseudo-second-order adsorption rate constants, *k*_1_ (min^−1^) and *k*_2_ (g mg^−1^ min^−1^), and adsorption capacity per unit of adsorbent mass at equilibrium, *q_e_* (mg g^−1^), by SWCNT BPs and 75% GO-SWCNT BPs for the three different NSAIDs (50 ppm).

	Pseudo-First Order Kinetics			Pseudo-Second Order Kinetics		
**Diclofenac**	***k*** **_1_ × ** **10^3^** **(min^−1^)**	***q_e_*** **(mg g^−1^)**	***R*** **^2^**	***k*** **_2_ × ** **10^4^** **(g mg^−1^ min^−1^)**	***q_e_*** **(mg g^−1^)**	***R*** **^2^**
SWCNT BP	10.1 ± 1.1	96.4 ± 2.5	0.9823	2.6 ± 0.4	103.0 ± 5.4	0.9961
75% GO-SWCNT BP	32.5 ± 2.5	116.1 ± 2.0	0.9752	8.4 ± 1.8	120.5 ± 8.3	0.9914
**Ketoprofen**	***k*** **_1_ × ** **10^3^** **(min^−1^)**	***q_e_*** **(mg g^−1^)**	***R*** **^2^**	***k*** **_2_ × ** **10^3^** **(g mg^−1^ min^−1^)**	***q_e_*** **(mg g^−1^)**	***R*** **^2^**
SWCNT BP	6.1 ± 0.7	110.2 ± 3.0	0.9756	1.4 ± 0.3	118.7 ± 8.3	0.9939
75% GO-SWCNT BP	6.4 ± 0.7	112.8 ± 3.1	0.9777	1.5 ± 0.3	121.8 ± 8.8	0.9934
**Naproxen**	***k*** **_1_ × ** **10^3^** **(min^−1^)**	***q_e_*** **(mg g^−1^)**	***R*** **^2^**	***k*** **_2_ × ** **10^3^** **(g mg^−1^ min^−1^)**	***q_e_*** **(mg g^−1^)**	***R*** **^2^**
SWCNT BP	2.9 ± 0.1	106.6 ± 0.8	0.9943	5.3 ± 1.1	121.1 ± 9.5	0.9980
75% GO-SWCNT BP	3.2 ± 0.1	125.9 ± 0.8	0.9960	5.7 ± 1.4	142.2 ± 9.4	0.9988

## Data Availability

Data are contained within the article.

## Sample Availability

Samples are available from the authors.
